# DNA methylation as a potential diagnosis indicator for rapid discrimination of rare cancer cells and normal cells

**DOI:** 10.1038/srep11882

**Published:** 2015-07-03

**Authors:** Xingyu Si, Yaoyao Zhao, Chengdui Yang, Sichun Zhang, Xinrong Zhang

**Affiliations:** 1 Department of Chemistry, Beijing Key Laboratory for Microanalytical Methods and Instrument, Tsinghua University, Beijing 100084 (China)

## Abstract

The global DNA methylation degree may be a ubiquitous and early biomarker to distinguish cancer cells from benign cells. However, its usefulness in clinical diagnosis was scarcely demonstrated, because the cancer cells isolated from patients were usually very rare. Even if 10 mL of peripheral blood was sampled from a patient, only tens of cancer cells could be isolated. So a method to quantify DNA methylation from small number of cells was needed to apply DNA methylation in clinical environment. In this study, we found that normal breast cell line MCF10A and breast cancer cell line MCF7 cells present significantly different percentage of genomic 5-methylcytosine (p < 0.02, n = 8), it could be a potential indicator for rapid discrimination of rare cancer cells from normal cells. However, conventional mass spectrometry needs usually ~10^6^ cells to quantify DNA methylation degree, which was too large to be applied in clinical diagnosis. Here we developed a fast mass spectrometry-based method capable of analyzing the DNA methylation degree from only ~100 human cells. Our method could reveal the different DNA methylation degree between MCF10A and MCF7 cells in less than two hours, having the potential to provide reliable information for clinical application.

The technology to isolate and identify rare tumor cells out of large number of normal cells has shown increasing clinically importance in cancer prognosis, early diagnosis of metastatic tumor and assessment of anticancer drugs, such as the isolation of disseminated tumor cells in the bone marrow and the enrichment of circulating tumor cells (CTCs) in the peripheral blood^1^,^2^,^3^,^4^. Organ-specific antigens could be used to capture a specific type of tumor cells, but for an unknown type of cancer at the early stage of tumorigenesis, organ-specific markers may lead to false-negative results^5^,^6^. Another strategy was to separate the tumor cells by epithelial antigens or other markers, but false-positive results may also be given because these antigens were not tumor-specific^5^. For instance, epithelial antigens were commercially employed to isolate and enumerate the rare circulating tumor cells (CTCs) in the blood^7^,^8^. However, CTCs could not be discriminated from circulating epithelial normal cells, which also express epithelial antigens on the cell membrane, have a similar size and density of CTCs and circulate in the bloodstream^3^,^5^,^9^,^10^,^11^. Briefly, due to the lack of ideal biomarkers which are expressed in all type of cancers but not in any normal tissue, the specific isolation of rare cancer cells by immune-mediated approaches remains challenging.

Cytosine methylation in genomic DNA is a common epigenetic modification which is involved in many biological processes including carcinogenesis^12^,^13^,^14^,^15^,^16^,^17^,^18^,^19^,^20^,^21^,^22^,^23^. Many studies have shown that global change of DNA methylation degree is a ubiquitous feature of a wide variety of cancers^12^,^15^,^17^,^18^. This phenomenon could be observed early in tumorigenesis and the degree of the abnormity would increase with tumor progression or the extent of malignancy^12^. The global DNA methylation degree would be a promising criterion to demonstrate the cells’ tumor nature for nearly all types of cancer, thus helping determine whether the isolated cells were cancer cells or not before knowing the cancer type.

However, DNA methylation was scarcely used in a clinical environment, because distinguishing between cancer and normal cells by the degree of DNA methylation faces several challenges. Firstly, the cancer cells were usually very rare, especially at the early stage of carcinogenesis. In the case of CTCs, the number of tumor cells in the human blood is very limited, which is about 1~10 cells per milliliter whole blood in average^24^. Such a small number of cells could not provide enough amount of DNA for current DNA methylation quantification methods, such as HPLC-based method or immunochemistry assay which usually need microgram amounts of DNA^25^,^26^,^27^. Secondly, current methods for global DNA methylation analysis were usually time and labor consuming, thus being unsuitable for the fast clinical diagnosis of cancers, because several tedious steps of sample pretreatment must be taken to obtain enough amount of pure DNA. Consequently, such a small number of the isolated cancer cells from the patients and the requirement for speed in clinical assay forced us to explore a sensitive, fast and convenient method capable of analyzing the methylation degree of such a small number of cells.

Herein we described a sensitive and fast nanoelectrospray ionization mass spectrometry (nanoESI-MS) based method to discriminate rare cancer cells and normal cells by determining their genomic DNA methylation degree, sampling only 100 human cells. Combining our method with conventional enrichment methods could avoid the false-positive results obtained by traditional immune-mediated methods, thus providing more reliable clinical information for cancer prognosis and early diagnosis of tumor.

## Results and Discussion

**Figure 1:**
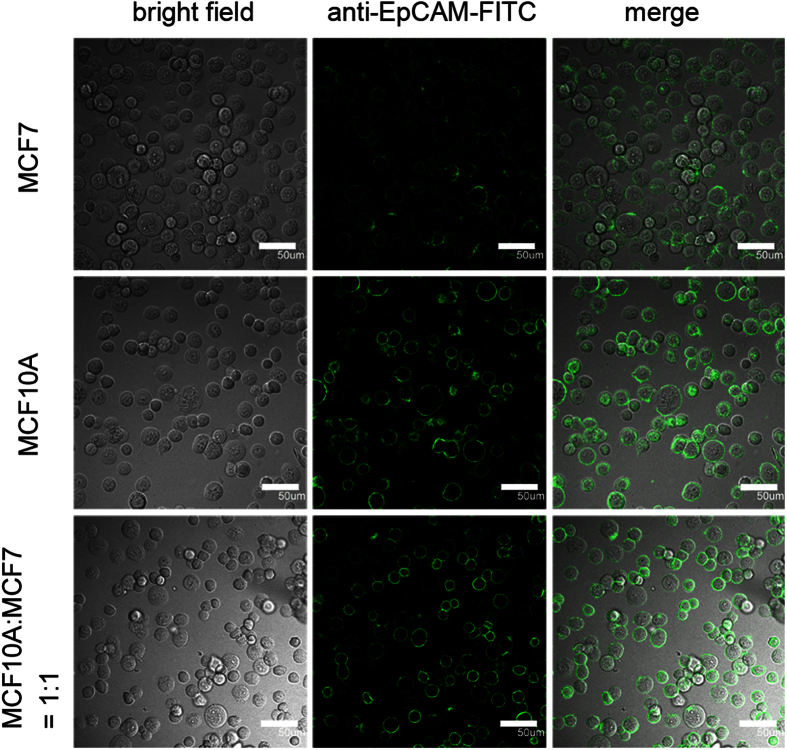
MCF7, MCF10A and the mixture of both cells were stained by EpCAM antibodies conjugated to FITC. Scale bar = 50 μm.

Although immuno-mediated approaches were widely applied for the isolation and identification of cancer cells, the false-positive results might be obtained as indicated in our following experiment. Two cell lines were selected to represent the normal cells and cancer cells: One was normal breast cell line MCF10A, the other one was breast cancer cell line MCF7. The cells of either cell line and the mixture of both cells were incubated with EpCAM antibodies conjugated to FITC (Fig. 1). The merged images of bright field and dark field obtained by fluorescence microscopy had shown that the cell membrane of each cell on the focal plane from both cell lines could be stained by anti-EpCAM-FITC, suggesting that EpCAM was expressed on the cell membrane of both cell lines (Fig. 1). The two cell lines could not be distinguished from each other in the dark image of the cell mixture. Since EpCAM was a most frequently used epithelial antigen to isolate tumor cells from the bone marrow and the peripheral blood, this test suggested that epithelial normal cells and cancer cells could not be well distinguished by conventional methods based on epithelial antigens.

The results above are consistent with previous reports. For example, previous researches have used commercial antibodies specific to epithelial antigens for isolation of CTCs, thus enriching the benign epithelial cells and CTCs in the blood indiscriminately and giving false-positive results^5^,^9^,^10^,^11^. Several papers have found epithelial cells in the blood of subjects without malignancy, such as the patients with benign inflammatory colon disease^11^, benign bowel disease^28^, or epithelial proliferative disease^29^,^30^, and the subjects after the surgery of benign breast cancer^31^. These epithelial normal cells were detectable with current CTC assays, indicating the need for further characterization of the cells. Our experiment indicated that immune-mediated methods were not specific enough to isolate the cancer cells, calling for further characterization of the isolated cells.

To find a method for rapid discrimination of rare cancer cells and normal cells, the global DNA methylation degree was studied in our subsequent experiment, because tumor cells would present significantly different degree of methylation^12^,^17^,^18^. The DNA of ~10^6^ cells from MCF10A cell line or MCF7 cell line was hydrolyzed to bases according to the process reported by previous researches with minor modification^32^. Released cytosine (Cyt) and 5-methylcytosine (5mCyt) were resolved in 1 mL methanol without purification. The genomic DNA methylation degree was calculated by the relative signal of cytosine (Cyt) and 5-methylcytosine (5mCyt) obtained in ESI-MS analysis after proper correction. Results showed a significant difference of DNA methylation degree of MCF10A and MCF7 (p < 0.02, n = 8), demonstrating the robustness of discriminating normal cells and tumor cells by their DNA methylation degree (Table 1).

However, current methods to quantify DNA methylation degree by mass spectrometry were unsuitable for clinical assays, because the cancer cells isolated from the patients are usually very rare. We tried to analyze 100 MCF10A or 100 MCF7 cells by ESI-MS according to the procedure above. Unfortunately, even if smaller volume of solvent, such as 200 μL of methanol, was used to resolve the cell lysate, no signals of 5mCyt were obtained from the mass spectra (Data not shown). Usually, there is only 1~10 CTCs per milliliter whole blood in average, if 10 mL blood was sampled, there would be only 100 cells for the further characterization at most^24^. If we want to make DNA methylayion-based method available for most of the cancer patients, the method must handle at most 100 human cells. Current assays are still unable to deal with such a small number of cells.

**Figure 2:**
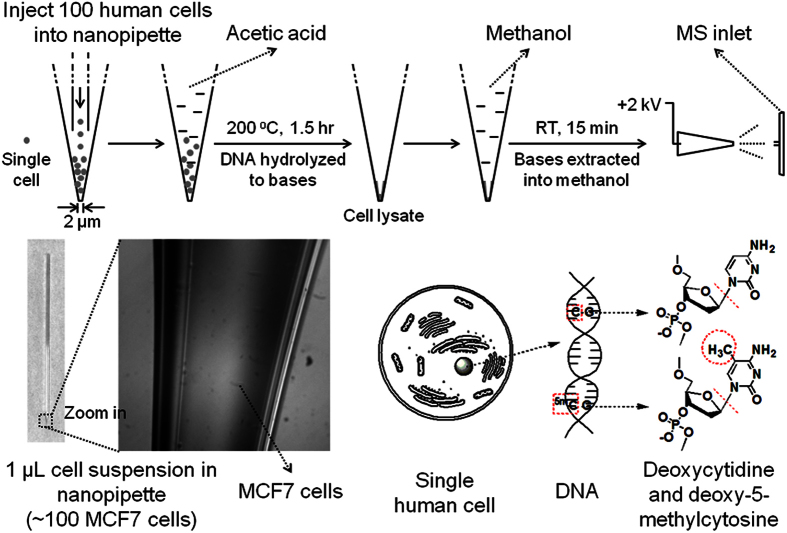
The procedure of in-pipette hydrolysis of 100 human cells and nanoelectrospray ionization was shown. By hydrolyzing genomic DNA to bases, deoxycytidine and deoxy-5-methylcytidine would become Cyt and 5mCyt, the molecular weights of which were 111 and 125 respectively. Thus, they could be discriminated by mass spectrometer, indicating the methylation degree of genomic DNA. 100 MCF7 cells in water were injected in the tip of a nanopipette before being hydrolyzed.

**Figure 3:**
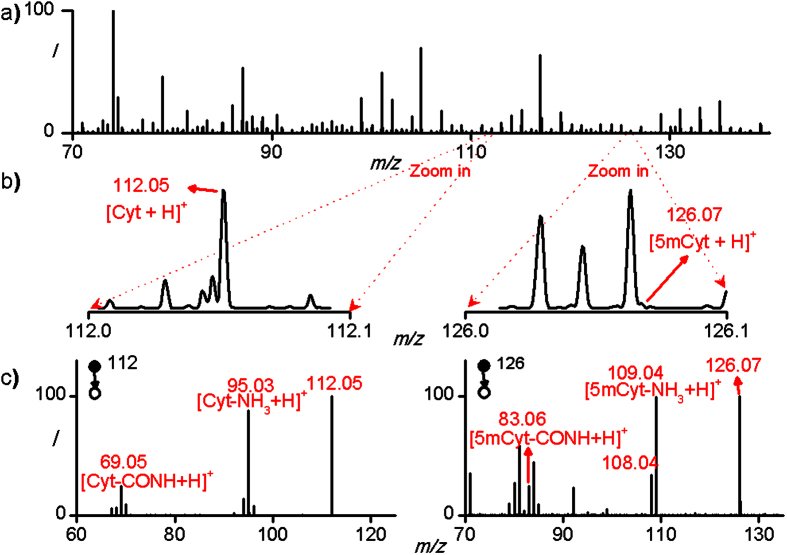
**a**) Mass spectrum of 100 hydrolyzed MCF7 cells in positive-ion mode averaged by 20 scans. **b**) Zoomed mass spectrum of 100 MCF7 cells showing the peaks of [Cyt+H]^+^ and [5mCyt+H]^+^. ^c^) MS^2^ spectra of [Cyt+H]^+^ and [5mCyt+H]^+^ averaged by 20 scans.

To achieve the goal above, we develop a simple and fast method capable of determining the genomic DNA methylation degree of ~100 human cells by directly introducing a hydrolysis reaction into a nanopipette which also served as the spray emitter of nanoESI-MS, thus empowering nanoESI-MS to detect the Cyt and 5mCyt in only 100 human cells. From sampling to obtaining the mass spectra, the whole procedure only lasted for less than 2 hours, without DNA isolation, polymerase chain reaction or any other tedious steps (Fig. 2). Figure 3a shows a typical mass spectrum of 100 hydrolyzed MCF7 cells in positive-ion mode. Because of the complexity of the sample and the relatively low amount of Cyt and 5mCyt in 100 cells, the peaks of both [Cyt+H]^+^ and [5mCyt+H]^+^ were hard to found in MS spectra (m/z = 112 and 126 respectively) even if the spectrum was zoomed in, especially for [5mCyt+H]^+^ (Fig. 3b). However, when 112 and 126 were set as the parent ions, the peaks of [Cyt-NH_3_+H]^+^ and [5mCyt-NH_3_+H]^+^ could be found easily in MS^2^ speatra (m/z = 95 and 109 respectively) (Fig. 3c). Besides, [Cyt-CONH+H]^+^ and [5mCyt-CONH+H]^+^ could also be found (m/z = 69 and 83 respectively). The accurate values of m/z of these four peaks were exactly the same as those of Cyt and 5mCyt standards, identifying Cyt and 5mCyt in cell lysate (Figure S1).

To improve the speed of our method, conventional steps of DNA extraction and purification were both avoided. Cell lysis and DNA hydrolysis were integrated into one step. In addition, to improve the sensitivity and reduce the sample loss, the DNA from 100 cells was directly hydrolyzed in the spray emitter of nanoESI-MS, thus saving the sample to the greatest extent (Fig. 2). Besides, our method could avoid the matrix effects caused by the complexity of the sample^33^, even if sample purification was not conducted, because 1) some matrix, such as salts, could not dissolve into pure methanol, resulting in some tolerance of matrix effects; 2) single reaction monitoring (SRM) was always utilized to identify Cyt and 5mCyt in MS^2^ spectra. Therefore, SRM were always used to identify Cyt and 5mCyt while the samples were ionized.

**Figure 4:**
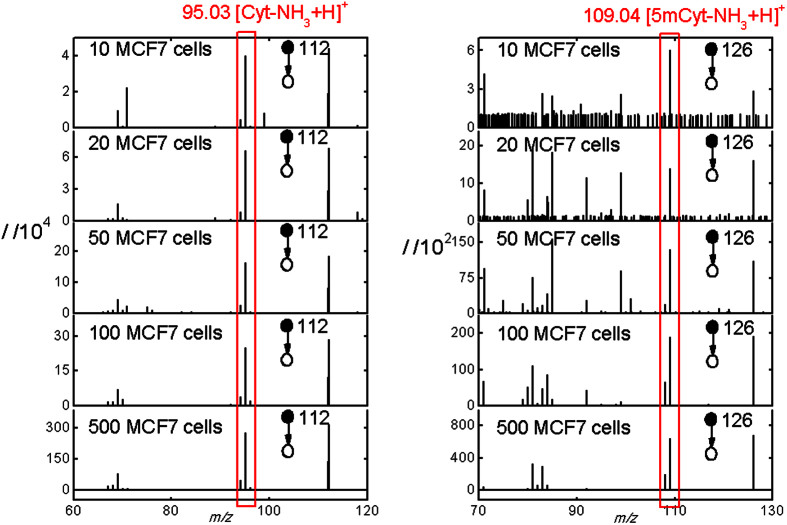
MS^2^ spectra of [Cyt+H]^+^ and [5mCyt+H]^+^ when sampling different numbers of MCF7 cells, averaged by 20 scans. At least 100 human cells were needed to obtain stable signal of [5mCyt-NH_3_+H]^+^ which was long enough to calculate the methylation degree.

The time of extraction and the solution for nanoelectrospray were all optimized to maximize the intensities of both [Cyt-NH_3_+H]^+^ and [5mCyt-NH_3_+H]^+^ (Figure S2). As a result, the signal of either [Cyt-NH_3_+H]^+^ or [5mCyt-NH_3_+H]^+^ of as less as 10 MCF7 cells could be detected by our method (Fig. 4). But the duration of the signal was too short to collect the intensities of both ions. Therefore, at least 100 human cells were needed to obtain stable signal of [5mCyt-NH_3_+H]^+^ which was long enough to calculate the DNA methylation degree. The relative standard deviation for 100 MCF7 cells and 100 MCF10A cells is 14% and 18% respectively (n = 7). The capability of analyzing such a limited number of cells means that our method could be applied for the analysis of the few isolated tumor cells.

Our method could reveal the different DNA methylation degree not only for 100 MCF10A and MCF7 cells (1.10% and 0.93% respectively), but also for other cell types such as HepG2 and Hela cells (1.12% and 1.06% respectively). The high sensitivity and universality of our method implies that it may be applied for the analysis of other types of cancer except for breast cancer.

In conclusion, we proposed a novel strategy to discriminate the rare normal cells and cancer cells by their genomic DNA methylation degree. The method we presented here combined conventional nanoESI-MS with direct in-pipette hydrolysis, realizing the rapid determination of genomic DNA methylation degree of only 100 human cells. Our method might be applied for the discrimination between few cancer and few normal cells, such as the discrimination of CTCs in the peripheral blood, due to several advantages including 1) simple and fast procedure, 2) saving the sample to the greatest extent, thus being sensitive enough to detect only 100 human cells, 3) universality to several human cell types, thus being able to distinguish between MCF10A and MCF7 cells at 100-cell level, making the identification of cancer cells more reliable.

### Experimental Section

#### Materials

Cytosine and 5-methylcytosine·HCl standards, methanol, ethanol, 2-propanol and acetonitrile were all purchased from Sigma-Aldrich. Acetic acid (AR) was purchased from Beijing Chemical Works. The water was purified using a filtration system (ThermoFisher Scientific). HepG2, Hela and MCF10A cells were purchased from National Platform of Experimental Cell Resources for Sci-Tech in China. MCF7 cells were purchased from ABGENT. The culturing medium, trypsin-EDTA, PBS and other materials used in cell culturing were all purchased from Corning (NY, USA). The EpCAM antibodies conjugated to FITC were purchased from Miltenyi Biotec Incorporation.

The nanopipettes used as the emmiter for nanoESI-MS were ~5 cm long with a tip of 2-μm diameter (Fig. 2). These nanopipettes were pulled from borosilicate glass capillaries (length = 10 cm, i.d = 0.59 mm, o.d. = 1.0 mm, Vitalsense Scientific Instruments Co., Ltd.) by a P-2000 laser puller (Sutter Instrument Co.) with a program of following parameters: Heat = 300, Fil = 5, Vel = 30, Del = 128, and Pul = 70.

#### Cell culture and harvesting

MCF7, HepG2 and Hela cells were grown in DMEM supplemented with 10% fetal bovine serum (FBS) and 1% penicillin/streptomycin in a humidified incubator containing 5% CO_2_ at 37 °C. MCF10A cells were grown in DMEM/F12 (1:1) supplemented with 5% horse serum, 10 μg·L^−1^ insulin, 20 mg⋅L^−1^ epidermal growth factor, 100 ng·L^−1^ cholera toxin and 1% penicillin/streptomycin. To harvest the cells, 1 mL trpsin/EDTA was added into a 6-cm cell plate containing ~10^6^ cells. After 3-minute incubation at room temperature, the 1 mL cell suspension was transferred into a 1.5 mL Eppendorf tube. The suspension was centrifuged at 2000 rpm for 10 minutes to remove the supernatant containing trpsin/EDTA.

#### Determining DNA methylation degree using large number of cells

Harvested MCF10A or MCF7 cells were suspended in 1 mL 0.9% NaCl water solution. 0.5–1.0 × 10^6^ cells were transferred to a new Eppendorf tube. The suspension was centrifuged at 2000 rpm for 10 minutes to remove the supernatant containing NaCl. Afterwards, 1 mL formic acid was added into the tube to suspend the cells. The whole suspension was transferred into a 1.5 mL glass vial. The vial was heated in a muffle furnace at 140 °C for 1.5 hours. Then the residual formic acid was evaporated in a vacuum drier at 60 °C until the cell hydrolysate was totally dried. 1 mL methanol was added into the vial to resolve the Cyt and 5mCyt in the lysate. The sample was directly analyzed by commercial ESI-MS (Orbitrap mass spectrometer, Q-Exactive, Thermo Scientific, San Jose, CA). The DNA methylation degree was directly calculated by the intensities of [Cyt+H]^+^ and [5mCyt+H]^+^ in the MS spectrum according to the standard curve made by analyzing a series of Cyt or 5mCyt standards dissolved in methanol.

#### Immunofluorescent staining of MCF10A and MCF7 cells

The immunofluorescent staining of MCF10A and MCF7 cells was conducted according to the manufacturer’s protocol with minor modification. Harvested MCF10A and MCF7 cells were centrifuged at 2000 rpm for 10 minutes to remove the supernatant containing trpsin/EDTA. The cells were resusupended in 1 mL DPBS and counted. The 1:1 mixture of both cells was made according to the concentration of both cell suspensions. Afterwards, the cell suspensions of MCF10A, MCF7 and the mixture were centrifuged at 2000 rpm for 10 minutes to remove the supernatant. The cells were resuspended in 100 μL buffer containing PBS, 0.5% bovine serum albumin (BSA), and 2 mM EDTA. 10 μL of anti-EpCAM-FITC was added. The suspensions were mixed well and incubated for 10 minutes in the dark in the refrigerator (4 °C). Then 1 mL buffer was added into the suspensions. The suspensions were centrifuged at 2000 rpm for 10 minutes to aspirate the supernatant completely. The stained cell pellet was resuspended in a suitable amount of buffer for analysis by fluorescence microscopy (Olympus IX81 confocal microscope).

#### Hydrolyzing few cells in a nanopipette

Harvested cells were diluted by water to a final concentration of 1 × 10^5^ cells·mL^−1^. 1 μL of such a suspension containing ~100 human cells was injected into the tip of a nanopipette by a homemade micropipette. ~15 μL acetic acid was soon injected into the nanopipette. Then the end of the nanopipette was sealed by some silica glue. Afterwards, the nanopipette was heated in a muffle furnace at 200 °C for 1.5 hours, and then cooled down for several minutes. All of the acetic acid would be used up at the end of hydrolysis. Before analyzed by MS, the Cyt and 5mCyt in the cell hydrolysate would be extracted into ~15 μL methanol added into the nanopipette for 15 minutes.

#### Mass spectrometry analysis

All the mass spectra were recorded by an Orbitrap mass spectrometer (Q-Exactive, Thermo Scientific, San Jose, CA). Capillary temperature: 320 °C; tube lens voltage: 50 V; mass resolution: 35000; maximun inject time: 50 ms; microscans: 1. For analyzing large number of cells, the sample was directly ionized by the commercial ESI source. For analyzing ~100 human cells, the commercial ESI source was removed and the nanopipette containing the sample would be placed in front of the ion transfer tube. The distance between the tip of the nanopipette and the MS inlet was ~5 mm. A home-built power supply provided a positive electric voltage of 2 kV. A copper wire was inserted into the nanopipette so that the voltage can be applied to the spray solution.

Ahead of the analysis, the scan type of the mass spectrometer was selected as AIF-MS/MS mode. Center: 112.05 for Cyt or 126.05 for 5mCyt; width = 0.4; charge = 1; NCE = 80 eV. After the electric was applied on the sample, the parent ion would be changed between 112 and 126 for at least 6 times.

#### Control experiments

Four controls were made to prove that the hydrolysis reaction did happen (Figure S3). Firstly, pure methanol was directly analyzed by nanoESI-MS as the background of the spray solution. Secondly, ~15 μL acetic acid was injected into an empty nanopipette, sealed and heated at 200 °C for 1.5 hours. ~15 μL methanol was added and the nanopipette was placed at room temperature for 15 minutes. Then the sample was analyzed by nanoESI-MS to show the background of the condensed acetic acid. Thirdly, 1 μL suspension containing 100 MCF7 cells was injected into the tip of a nanopipette, placed at room temperature for 5 hours until the suspension was dried. Then ~15 μL methanol was added and the nanopipette was placed at room temperature for 15 minutes. The sample was analyzed by nanoESI-MS to prove that the hydrolysis of the cells was prerequisite to the detection of Cyt and 5mCyt. Fourthly, 100 MCF7 cells were hydrolyzed according to the steps described above except that the acetic acid was replaced by ethanol. Finally, 100 MCF7 were hydrolyzed and analyzed just as the procedure described above in comparison with the other four controls.

#### Assay optimization

All the optimization experiments were carried out using 500 MCF7 cells (n = 5). Firstly, cells were hydrolyzed at 160 °C for different time, extracted by ethanol for 5 minutes. Taking both the sensitivity and the speed of our method into consideration, we chose 1.5 hours as a proper time for hydrolysis. Secondly, different temperature of hydrolysis was tried and 200 °C was chosen finally. Thirdly, time of extraction was optimized to be 15 minutes. Fourthly, different solution for both extraction and spray was tried. Among all the solutions, methanol showed the best performance.

#### Calculating methylation degree

Two standard curves were made by analyzing a series of Cyt or 5mCyt standards dissolved in methanol. The standard solution was supplemented with 5 mmol·L^−1^ ammonium acetate to simulate the buffering environment of the cell lysate. The DNA methylation degree was calculated by the relative concentration of [Cyt-NH_3_+H]^+^ and [5mCyt-NH_3_+H]^+^ according to the standard curve.

## Additional Information

**How to cite this article**: Si, X. *et al.* DNA methylation as a potential diagnosis indicator for rapid discrimination of rare cancer cells and normal cells. *Sci. Rep.* **5**, 11882; doi: 10.1038/srep11882 (2015).

## Supplementary Material

Supplementary Information

## Figures and Tables

**Table 1:** The global DNA methylation degree of MCF7 and MCF10A cells.

Group	MCF7			MCF10A		
	Cyt/μmol·L^−1^	5mCyt/nmol·L^−1^	% of 5mCyt^a^	Cyt/μmol·L^−1^	5mCyt/nmol·L^−1^	% of 5mCyt^a^
1	0.775 ± 0.064	9.34 ± 0.77	1.191 ± 0.011	1.463 ± 0.144	17.55 ± 1.84	1.185 ± 0.011
2	0.647 ± 0.003	7.70 ± 0.15	1.177 ± 0.024	1.098 ± 0.058	11.87 ± 0.66	1.069 ± 0.015
3	1.851 ± 0.035	21.85 ± 0.28	1.167 ± 0.022	2.076 ± 0.081	23.55 ± 0.93	1.122 ± 0.001
4	2.033 ± 0.096	24.22 ± 1.31	1.177 ± 0.008	1.962 ± 0.209	23.06 ± 2.23	1.162 ± 0.016
5	1.611 ± 0.090	18.80 ± 1.02	1.153 ± 0.007	1.288 ± 0.037	14.38 ± 0.34	1.104 ± 0.016
6	0.977 ± 0.025	11.58 ± 0.18	1.173 ± 0.014	2.545 ± 0.028	28.55 ± 0.75	1.109 ± 0.022
7	1.746 ± 0.064	20.60 ± 0.75	1.166 ± 0.029	1.730 ± 0.095	19.63 ± 1.15	1.122 ± 0.016
8	2.283 ± 0.606	26.96 ± 7.39	1.166 ± 0.021	2.483 ± 0.638	28.85 ± 7.70	1.147 ± 0.013
Averaged			1.171 ± 0.011			1.128 ± 0.036
Significance	p < 0.02, n = 8^b^					

^a^The % of 5mCyt = 5mCyt × 100 / (5mCyt+Cyt).

^b^The significant difference of % of 5mCyt of MCF7 and MCF10A cell lines.
