# Supplementary material for: DNA methylation as a potential diagnosis indicator for rapid discrimination of rare cancer cells and normal cells
Source: Sci Rep. 2015 Jul 3;5:11882. doi: 10.1038/srep11882 (PMC5155617; doi:10.1038/srep11882)
Supplement: Supplementary Information [file srep11882-s1.pdf]

Supporting information

DNA methylation as a potential diagnosis indicator for rapid discrimination of rare cancer cells and normal cells

Xingyu Si, Yaoyao Zhao, Chengdui Yang, Sichun Zhang, and Xinrong Zhang*

Supplementary discussion

Hydrolyzing 100 cells in the nanopipette was a crucial step in our method, because the nanopipette was not only a micro-reactor but the emitter for electrospray, avoiding the waste of sample to the greatest extent. The success in hydrolyzing cells in the nanopipette also means that our method suggests a potential application for conducting a variety of reactions on small-volume biological sample prior to sample detection. In order to demonstrate that the hydrolysis reaction did happen, several controls were made (Figure S3). When the 1 μL suspension containing 100 MCF7 cells was just evaporated in the nanopipette at room temperature for 5 hours, without acetic acid being added, the cells would also condense at the tip of the nanopipette. But even if the other steps were the same, the intensity of either $[Cyt\text{-}NH_3+H]^+$ or $[5mCyt\text{-}NH_3+H]^+$ would be relatively low (Figure S3). When the acetic acid was replaced by ethanol, whose acidity was much weaker than that of acetic acid, the intensity of either $[Cyt\text{-}NH_3+H]^+$ or $[5mCyt\text{-}NH_3+H]^+$ became higher than before, suggesting the hydrolysis of DNA did happen to some degree. But the intensity of either ion was still lower than that when acetic acid was used, indicating the intensity of either $[Cyt\text{-}NH_3+H]^+$ or $[5mCyt\text{-}NH_3+H]^+$ was associated with the acidity of the solution. In other words, the hydrolysis of DNA did happen as we designed and both ions detected mainly came from the hydrolysis of cells, instead of other sources.

Figure S4 shows the total ion current and extracted ion current intensity recorded by mass spectrometer when 100 hydrolyzed MCF7 cells were ionized. m/z = 112 or 126 were selected as parent ion alternatively. Usually, the signal of either $[Cyt\text{-}NH_3+H]^+$ or $[5mCyt\text{-}NH_3+H]^+$ could last for more than 1 minute if 100 human cells were hydrolyzed. During the first one minute after the electric voltage was applied, the signals of both ions were always stable. Therefore, during the first one minute, the parent ion was changed between 112 and 126 several times, and the intensities of both ions were recorded in turn. The DNA methylation degree was calculated by the relative intensity of $[Cyt\text{-}NH_3+H]^+$ and $[5mCyt\text{-}NH_3+H]^+$ after proper correction.

In order to show that our technique was also suitable for a mixed sample containing various population of both MCF10A and MCF7, we mixed MCF10A and MCF7 cells by five different ratios (100:0, 75:25, 50:50, 25:75 and 0:100), and measure their DNA methylation degrees with only 100 cells sampled (Figure S5). Results showed that they presented different methylation degrees, which correlated with the ratios of MCF7 versus MCF10A cells. Therefore, by measuring the averaged methylation degree of the 100 cells, the ratio of two types of cells could be preliminarily estimated. It is probable that both the normal epithelial cells and cancer cells circulate simultaneously in the bloodstream, thus being isolated together by conventional separation methods such as the commercial CellSearch system based on epithelial antigens. By conducting our method, the ratio of normal cells versus cancer cells could be estimated according to the averaged methylation degree, thus providing more detailed information for clinical use.

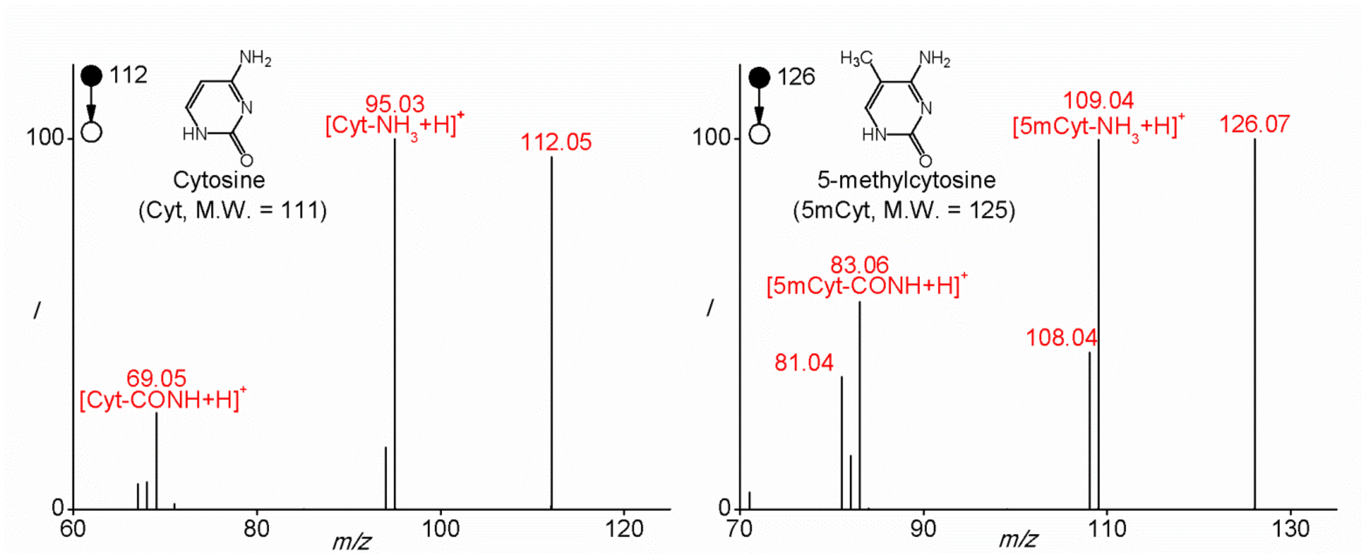

Figure S1. MS^2 spectra of $[Cyt+H]^+$ and $[5mCyt+H]^+$ standards averaged by 20 scans, recorded when 1 mmol/L Cyt and 5mCyt in ethanol were analyzed by conventional nanoESI-MS. AIF-MS/MS mode: Center = 112.05 for Cyt or 126.05 for 5mCyt, Width = 0.4, Charge = 1, NCE = 80 eV.

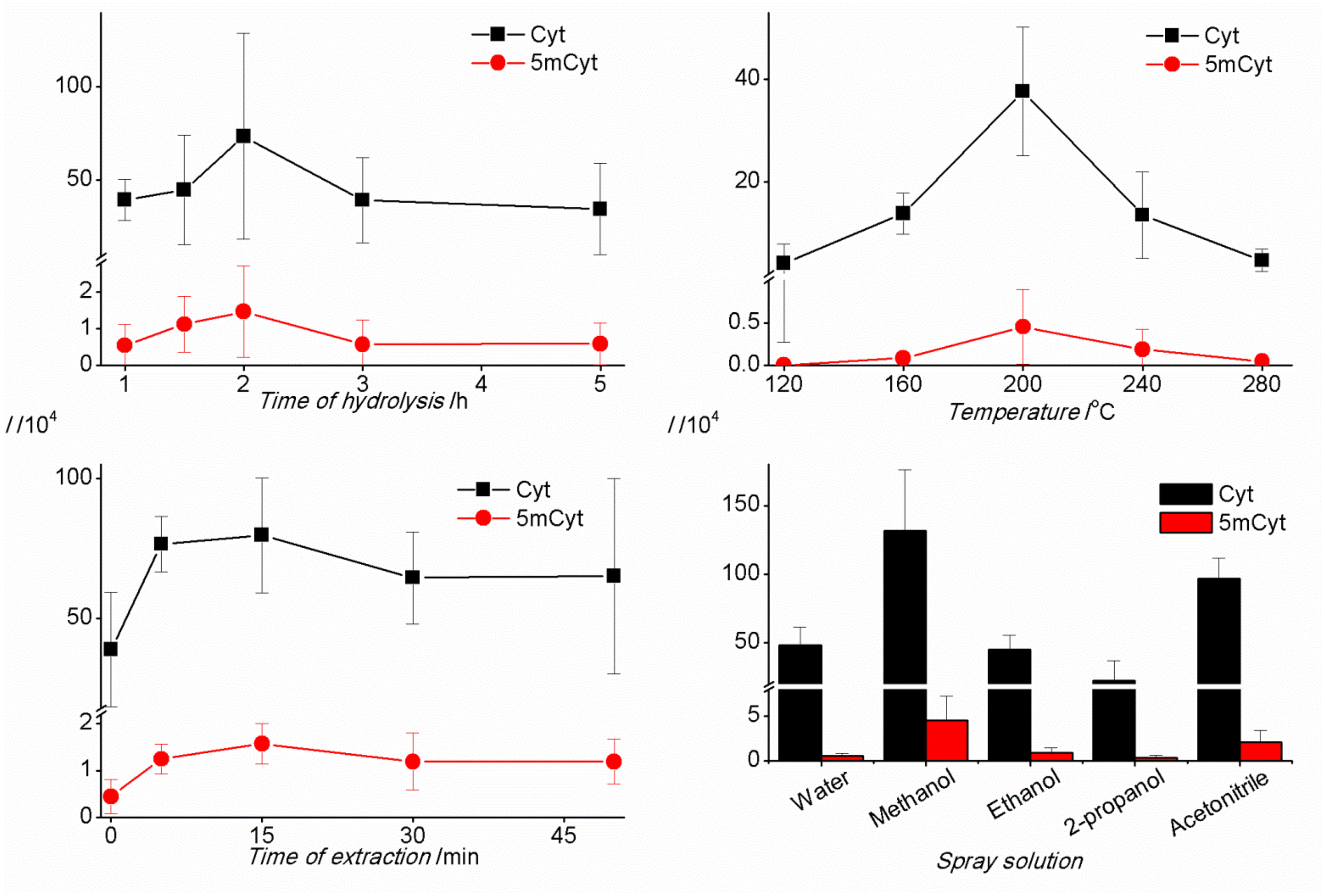

Figure S2. Optimization experiments. The intensities of both $[Cyt\text{-}NH_3+H]^+$ and $[5mCyt\text{-}NH_3+H]^+$ obtained under different conditions (n=5). The time and temperature of hydrolysis, the time of extraction and the solution for nanoelectrospray were all optimized. As a result, the cells were hydrolyzed at 200 °C for 1.5 hours and extracted by methanol for 15 minutes before being ionized.

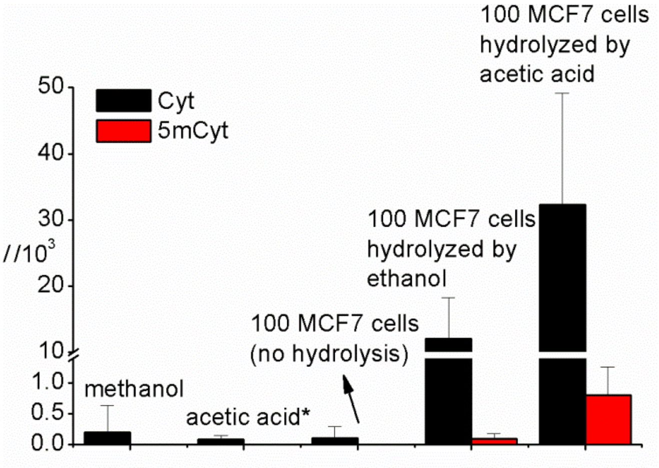

Figure S3. Control experiments. The averaged intensity of $[Cyt\text{-}NH_3+H]^+$ or $[5mCyt\text{-}NH_3+H]^+$ after different treatments (n=5). If 100 MCF7 cells were injected into the nanopipette but not hydrolyzed, the intensity of $[Cyt\text{-}NH_3+H]^+$ or $[5mCyt\text{-}NH_3+H]^+$ in SRM mode were just as low as those of them in pure methanol. On the other hand, if the cells were hydrolyzed by ethanol or acetic acid, the intensity of both ions would boost a lot, with an even higher intensity if acetic acid was used. *~15 μL acetic acid was injected into an empty nanopipette without cells being added. The other steps were the same as those when 100 MCF7 cells were hydrolyzed by acetic acid.

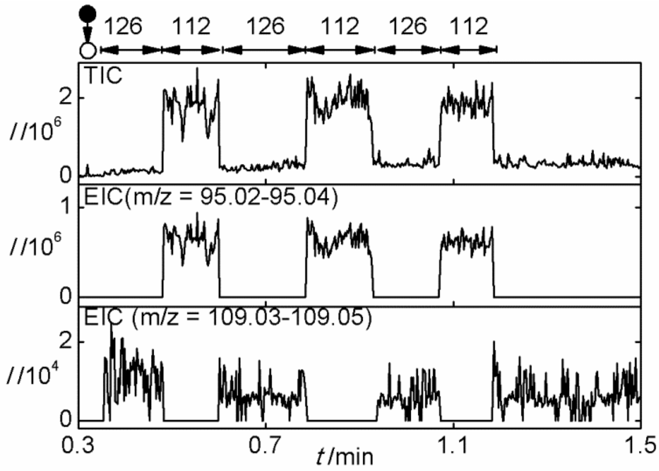

Figure S4. The total ion current (TIC) intensity and the extracted ion current (EIC) of $[Cyt\text{-}NH3+H]^+$ and $[5mCyt\text{-}NH3+H]^+$ recorded when 100 MCF7 cells were hydrolyzed. $[Cyt+H]^+$ and $[5mCyt+H]^+$ were selected as parent ion alternatively from the beginning of ionization. Usually, the signal could last for several minutes. The intensity of $[Cyt\text{-}NH3+H]^+$ and $[5mCyt\text{-}NH3+H]^+$ were obtained from the stable zone in TIC to calculate the DNA methylation degree.

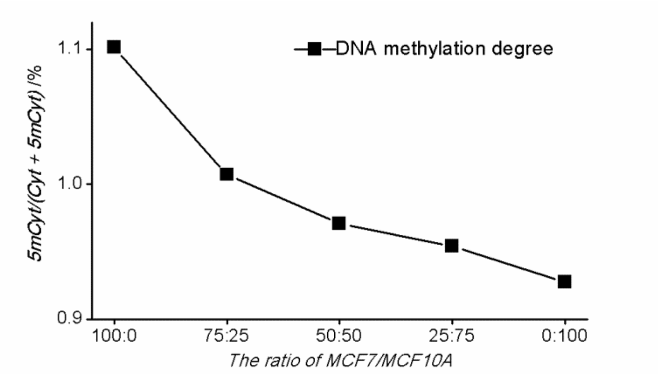

Figure S5. The DNA methylation degree of 100 mixed cells (pure MCF7, 75:25, 50:50, 25:75 of MCF7/MCF10A, pure MCF10A) calculated by our method.
